# Multiparametric MRI radiomics for noninvasive prediction of HER2 status in immunohistochemical 2+ breast cancer

**DOI:** 10.3389/fonc.2025.1765392

**Published:** 2026-01-14

**Authors:** Xiaoguang Li, Qiujie Dong, Hong Guo, Chunlai Zhang, Jing Tian, Cheng Cheng, Peng Zhong, Shan Gui, Chao Cong, Yi Wang

**Affiliations:** 1 Department of Radiology, Daping Hospital, Army Medical University, Chongqing, China; 2 Department of Nuclear Medicine, Daping Hospital, Army Medical University, Chongqing, China; 3 Department of Pathology, Daping Hospital, Army Medical University, Chongqing, China; 4 School of Electrical and Electronic Engineering, Chongqing University of Technology, Chongqing, China

**Keywords:** breast cancer, fluorescence *in situ* hybridization, human epidermal growth factor receptor 2, multiparametric magnetic resonance imaging, radiomics

## Abstract

**Background:**

Breast cancer (BC) patients with low expression of human epidermal growth factor receptor 2 (HER2), now classified as HER2-low, may benefit from new novel antibody-drug conjugates, such as trastuzumab deruxtecan. However, patients with immunohistochemistry (IHC) 2+ require further fluorescence *in situ* hybridization (FISH) to definitively determine HER2 status. Our study aims to explore the feasibility of radiomics models based on multiparametric magnetic resonance imaging (mpMRI) in predicting the HER2 status of IHC 2+ BC patients.

**Materials and methods:**

107 IHC 2+ BC patients, which composed of 65 (60.75%) FISH-negative and 42 (39.25%) FISH-positive, were enrolled in the retrospective study and divided into the training set (n = 74) and testing set (n = 33). The clinical and conventional MRI characteristics were used to build a clinical-imaging model. Radiomics features were extracted from T2-weighted imaging (T2WI), diffusion-weighted imaging (DWI), and DCE_phase3_ and DCE_phase7_ in the dynamic contrast-enhanced T1-weighted imaging (DCE-T1WI), and radiomics models were built based on each individual sequence, mpMRI sequences, and a combination of mpMRI sequences with clinical-imaging characteristics. The area under the receiver operating characteristic curve (AUC), sensitivity, specificity, and accuracy were calculated. DeLong test was used to determine the differences among various models. p-value <0.05 was considered statistically significant.

**Results:**

The clinical-imaging model achieved AUCs of 0.733 and 0.738 in the training and testing sets. Among the single-sequence radiomics models, the DCE_phase7_ model achieved the best predictive performance, with AUCs of 0.874 and 0.835 in the training and testing sets. The combined model C, composed of mpMRI model C (DCE_phase7_+DWI+T2WI) and clinical-imaging model, showed the best predictive performance, with AUCs of 0.952 and 0.892 in the training and testing sets.

**Conclusion:**

The mpMRI-based radiomics models had potential to noninvasively predict the HER2 status in IHC 2+ BC patients.

## Introduction

Breast cancer (BC) is a highly heterogeneous malignancy and the most commonly diagnosed cancer among women worldwide, posing a significant threat to women’s health (1). According to the expression of immunohistochemical biomarkers, including estrogen receptor (ER), progesterone receptor (PR), human epidermal growth factor receptor 2 (HER2), and Ki-67, BC can be classified into four molecular subtypes: luminal A, luminal B, HER2-overexpressing and triple-negative (2). These subtypes differ in treatment response, therapeutic effect evaluation, and clinical prognostic (3). HER2, a proto-oncogene member belonging to the epidermal growth factor receptor (EGFR) family, plays a key role in regulating cell growth, differentiation, and metastasis, and is frequently dysregulated in various tumors (4). HER2-positive BC accounts approximately for 10-20% (5). The overexpression of HER2 is strongly associated with malignant behaviors, poor prognosis, metastasis, and reduced overall survival (6). It also increases sensitivity to neoadjuvant chemotherapy and anti-HER2 therapy (7). Therefore, accurate determination of HER2 status is critical for guiding tailored treatment strategies and predicting prognosis in BC patients.

The clinical assessment of HER2 status primarily relies on immunohistochemistry (IHC) of biopsy specimens. According to the ASCO/CAP guidelines, IHC results are categorized as negative (0 or 1+), equivocal (2+), or positive (3+). Cases with an equivocal IHC results (2+) require further confirmation by fluorescence *in situ* hybridization (FISH) (8). The current diagnostic framework defines HER2-low as IHC 1+ or IHC 2+/FISH-negative, and HER2-positive as IHC 3+ or IHC 2+/FISH-positive (9). However, both IHC and FISH require invasive needle biopsy. Due to sampling limitations and intratumoral heterogeneity, biopsy may not fully represent the entire tumor ‘s characteristics. Furthermore, FISH testing is relatively costly and time-consuming, resulting in an increased medical burden for patients. Therefore, there is a clear need for a noninvasive, accurate, and cost-effective adjunctive tool to determine HER2 status in patients with IHC 2 +.

Radiomics is a rapidly emerging field for current precision medicine, which can extract and analyze large numbers of quantitative features from conventional medical images that may be imperceptible to the human eye and could be used to evaluate tumor heterogeneity (10). Breast multiparametric magnetic resonance imaging (mpMRI), including dynamic contrast enhanced T1-weighted imaging (DCE-T1WI), T2-weighted imaging (T2WI), and diffusion weighted imaging (DWI), is a well-established noninvasive method for breast cancer diagnosis (11). MRI-based radiomics has shown promising value in BC detection, aggressiveness evaluation, and survival analysis (12, 13). Therefore, this study aimed to explore the feasibility of mpMRI-based radiomics for predicting HER2 status in BC patients with equivocal IHC 2 +.

## Materials and methods

### Patients

**Figure 1:**
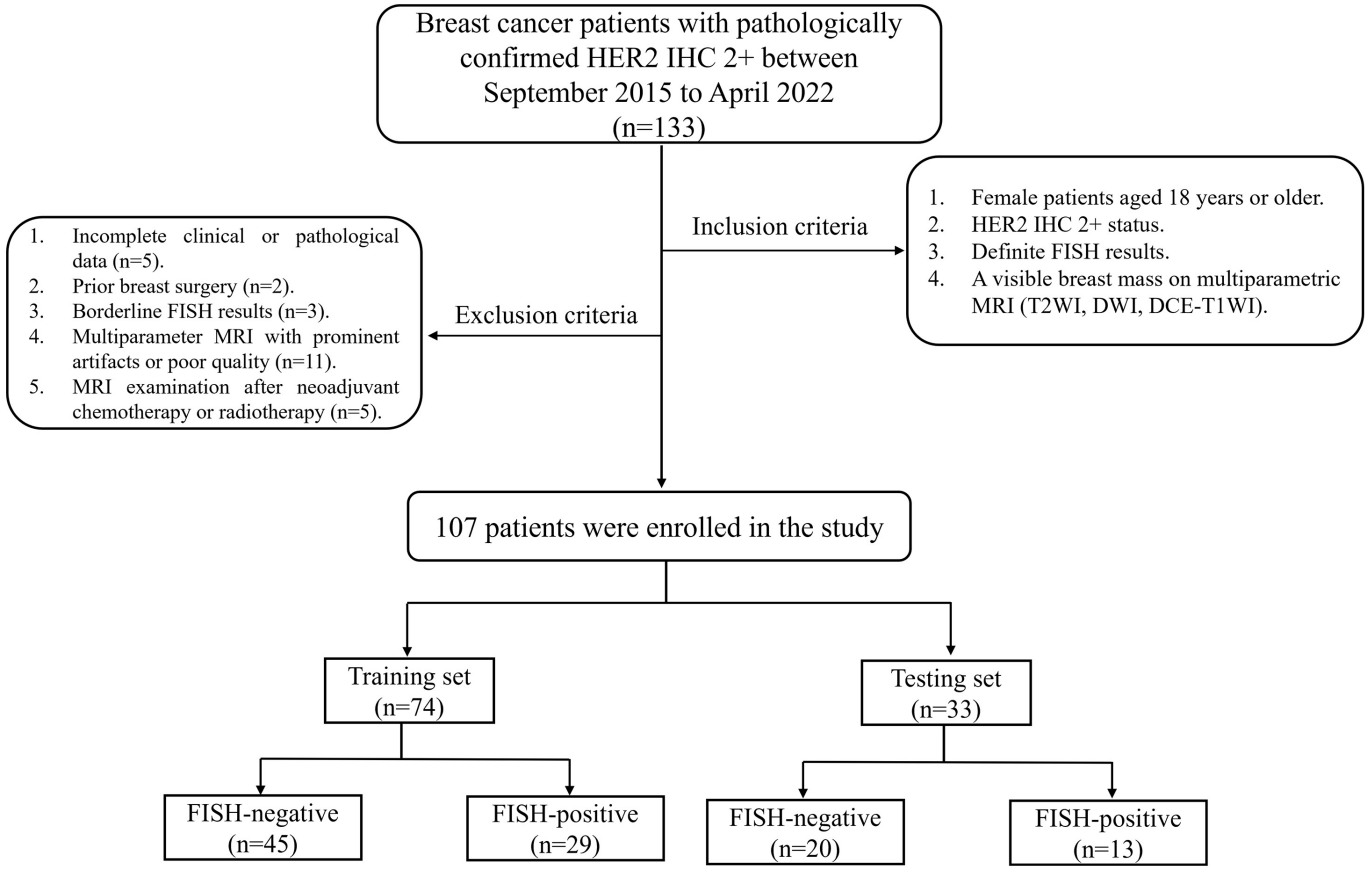
Study population flowchart.

This retrospective study was approved by our Institutional Review Board, which waived the requirement for informed consent [Ratification No: 2021(311)]. A total of 133 female BC patients with pathologically confirmed HER2 IHC 2+ who underwent breast mpMRI between September 2015 to April 2022 were retrospectively reviewed. Patients were screened from institutional pathology and imaging database. Inclusion criteria were: (1) age ≥ 18 years; (2) HER2 IHC 2+ status; (3) definite FISH results; (4) a visible breast mass on mpMRI (T2WI, DWI, DCE-T1WI). Exclusion criteria included: (1) incomplete clinical or pathological data (n=5); (2) prior breast surgery (n=2); (3) borderline FISH results (n=3); (4) mpMRI with prominent artifacts or poor quality (n=11); (5) MRI examination after neoadjuvant chemotherapy or radiotherapy (n=5). Finally, 107 patients (65 FISH-negative and 42 FISH-positive) were included and randomly divided into training (70.0%) and testing (30.0%) sets. The study flowchart was shown in **Figure 1**.

### MRI protocol

All MRI examinations were performed on a 1.5T scanner (MAGNETOM Aera, Siemens Healthcare, Erlangen, Germany) with a dedicated 8-channel breast coil. The mpMRI protocols included: (1) T1WI (TR = 8.6 ms, TE = 4.7 ms, FOV = 360 mm×360 mm, Matrix size=384×384, Pixel bandwidth=220, Flip angle=20°, Slice thickness=4.0 mm); (2) Fat-suppressed T2WI (TR = 5600 ms, TE = 57 ms, FOV = 340 mm×340 mm, Matrix size=320×320, Pixel bandwidth=250, Flip angle=142°,Slice thickness= 4.0 mm); (3) DWI (TR = 6300 ms, TE = 68 ms, FOV = 340 mm×159 mm, b=0, 50, 800, 1000 s/mm^2^, Matrix size=128×60, Pixel bandwidth=1700, Flip angle=90°, Slice thickness 4.0 mm); (4) DCE-T1WI (TR = 4.62 ms, TE = 1.75 ms, FOV = 360 mm×360mm, Matrix size=320×320, Pixel bandwidth=320, Flip angle=10°, Slice thickness=1.5 mm). DCE-T1WI was performed using the 3D volumetric interpolated breath-hold technique. For DCE-MRI, gadolinium-based contrast agent (Gd-DTPA, Magnevist, Bayer Healthcare, Berlin, Germany) was injected into the elbow vein by a high-pressure syringe at a flow rate of 2.5 ml/s, followed by a 15 mL saline flush at the same rate. A total of seven phases (one basal precontrast and six consecutive postcontrast phases) were collected, each phase scanning approximately 60 seconds, resulting a total of about 7 minutes.

### Image preprocessing, tumor segmentation, and feature extraction

Image preprocessing, tumor segmentation, and feature extraction were performed using 3D-Slicer (version 4.11.20210226, https://www.slicer.org/). T2WI, DWI (b = 1000 s/mm^2^), DCE_phase3_, and DCE_phase7_ images were retrieved from the Picture Archiving and Communication System and processed with the N4 Bias Field Correction module, followed by intensity normalization (0-2000) using Image Intensity Filter. After image preprocessing, manual three-dimensional region of interest (3D ROI) segmentation on all 107 cases was performed by radiologist 1 (with 10-year experience in breast imaging diagnosis). During the segmentation, ROIs were delineated layer by layer along the contour of the lesion. Given the importance of tumor heterogeneity, ROIs covered the entire lesion which included areas of hemorrhage, necrosis, and cyst across all sequences. To assess inter-observer reproducibility, Radiologist 2 (5 years of experience) independently segmented 35 randomly selected cases one month later. Both radiologists were blinded to clinical and pathological information.

Radiomics features were extracted using the PyRadiomics module. Prior to extraction, all images were resampled to isotropic voxels (1.0 × 1.0 × 1.0 mm^3^). Five Laplacian of Gaussian filters (σ=1-5) and a wavelet-based filter were used to process the original images. A total of 1,218 radiomics features could be obtained from each sequence, encompassing first-order, shape, gray level co-occurrence matrix (GLCM), gray level size zone matrix (GLSZM), gray level run length matrix (GLRLM), grey level dependence matrix features (GLDM), neighboring grey-tone difference matrix features (NGTDM), and wavelet features. All features comply with the Imaging Biomarker Standardization Initiative (IBSI). Features with an inter-class correlation coefficient (ICC) > 0.75 between the two radiologists were retained for subsequent analysis.

### Feature selection and model construction

**Figure 2:**
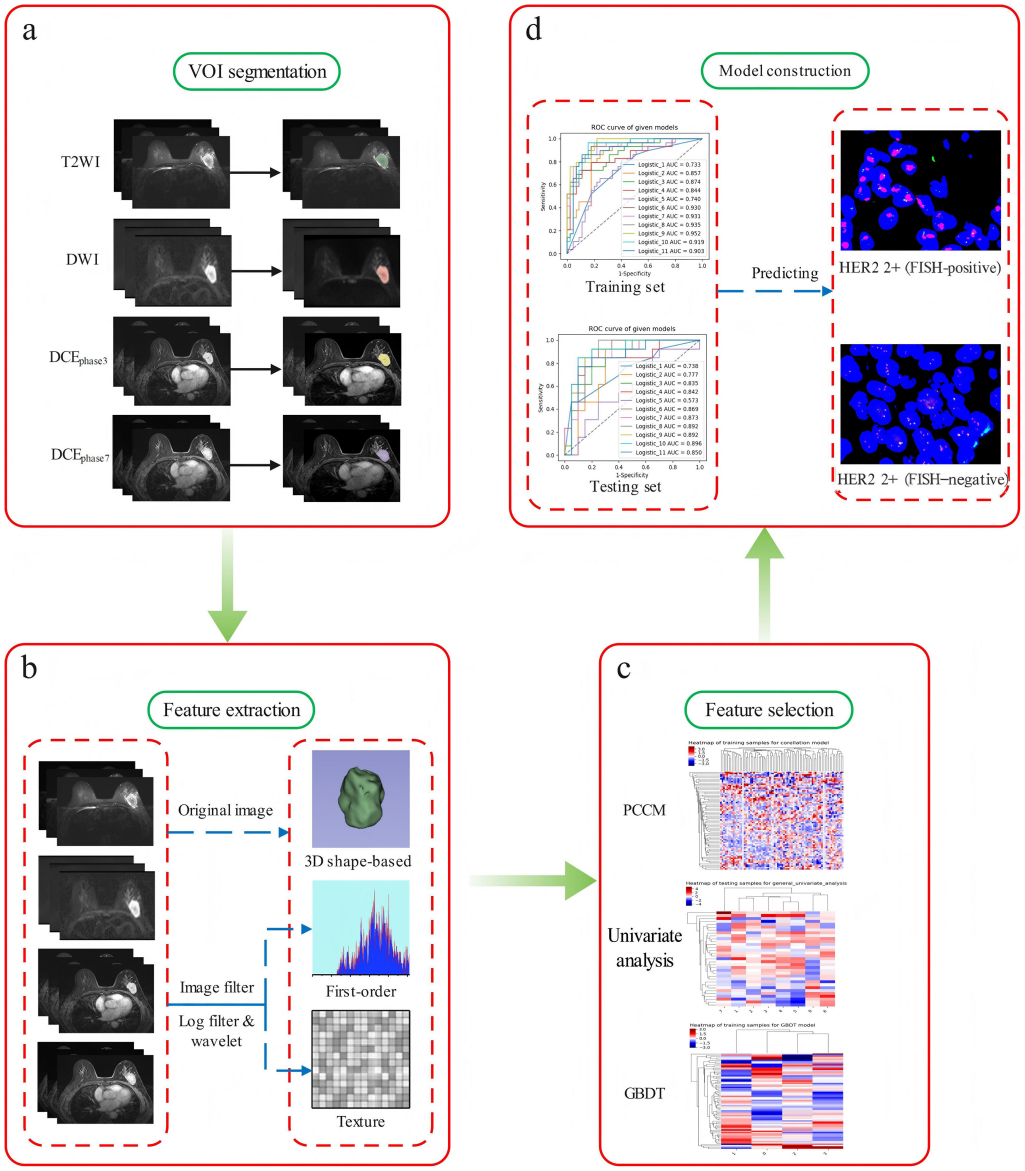
Overview of the radiomics workflow. **(a**: VOI segmentation; **b**: feature extraction; **c**: feature selection; **d**: model construction).

Radiomics features with ICC > 0.75 were selected and loaded into the GE IPMs platform (Version 2.5.2, GE Healthcare) for further processing. Missing values were imputed using the median, and all features were standardized z-score normalization to eliminate scale differences. We implemented a multi-step feature selection process to reduce feature dimensions and construct predictive models: First, Pearson’s correlation coefficient matrix (PCCM) was used to identify the collinearity between all the features. Features with a Pearson correlation coefficient > 0.80 were considered highly collinear. In such pairs, only the feature with higher univariate discriminative power was retained. Second, the univariate analysis was used to obtain features with statistically significant differences (p < 0.05) between the FISH-negative and FISH-positive groups. Third, the Gradient Boosting Decision Tree (GBDT) algorithm was performed to select the most predictive feature subset. Last, multivariate logistic regression models with 5-fold cross-validation were constructed based on the final selected features to establish both single-sequence and mpMRI radiomics models. The overall radiomics workflow was illustrated in **Figure 2**.

### Clinic and MRI characteristics and model construction

All pathological results were confirmed by two experienced pathologists. ER and PR were considered positive when more than 1% of cells exhibited nuclear staining (14). The Ki67 index ≥ 20% was considered high (15). Conventional MRI characteristics were independently assessed by two senior radiologists with 10 and 15-year experience in breast imaging diagnosis. Evaluated features included: maximum diameter, shape, margin, T2WI signal, cystic or necrosis component, peripheral edema, DCE_phase3_, DCE_phase7_ enhancement patterns, and TIC pattern. Any discrepancies in imaging features were resolved in agreement under the guidance of a third senior radiologists with 30 years of experience. For the clinical-imaging model, features with p-value < 0.05 in univariate logistic regression were entered into a multivariate logistic regression model with 5-fold cross-validation. In addition, combined models were built by integrating the selected mpMRI radiomics features with the significant clinical-imaging features.

### Statistical analysis

Statistical analyses were conducted using SPSS (version 24.0, SPSS Inc., Chicago, IL, USA), Medcalc (version 20.0, MedCalc Inc., Mariakerke, Belgium), and IPMs (Version 2.5.2, GE Healthcare) softwares. Data normality and homogeneity of variance were assessed with the Kolmogorov-Smirnov test and Levene’s test, respectively. Continuous data were presented as the means ± standard deviations (SD), and categorical variables as frequencies (proportions). Group comparisons were performed using independent t-tests or Mann-Whitney U tests for continuous variables, and chi-square or Fisher’s exact tests for categorical variables. Model performance was evaluated by the area under the receiver operating characteristic curve (AUC), sensitivity, specificity, and accuracy. AUC comparisons used the DeLong test. A two-sided p-value < 0.05 indicated statistical significance.

## Results

### Patient characteristics and clinical-imaging model

**Figure 3:**
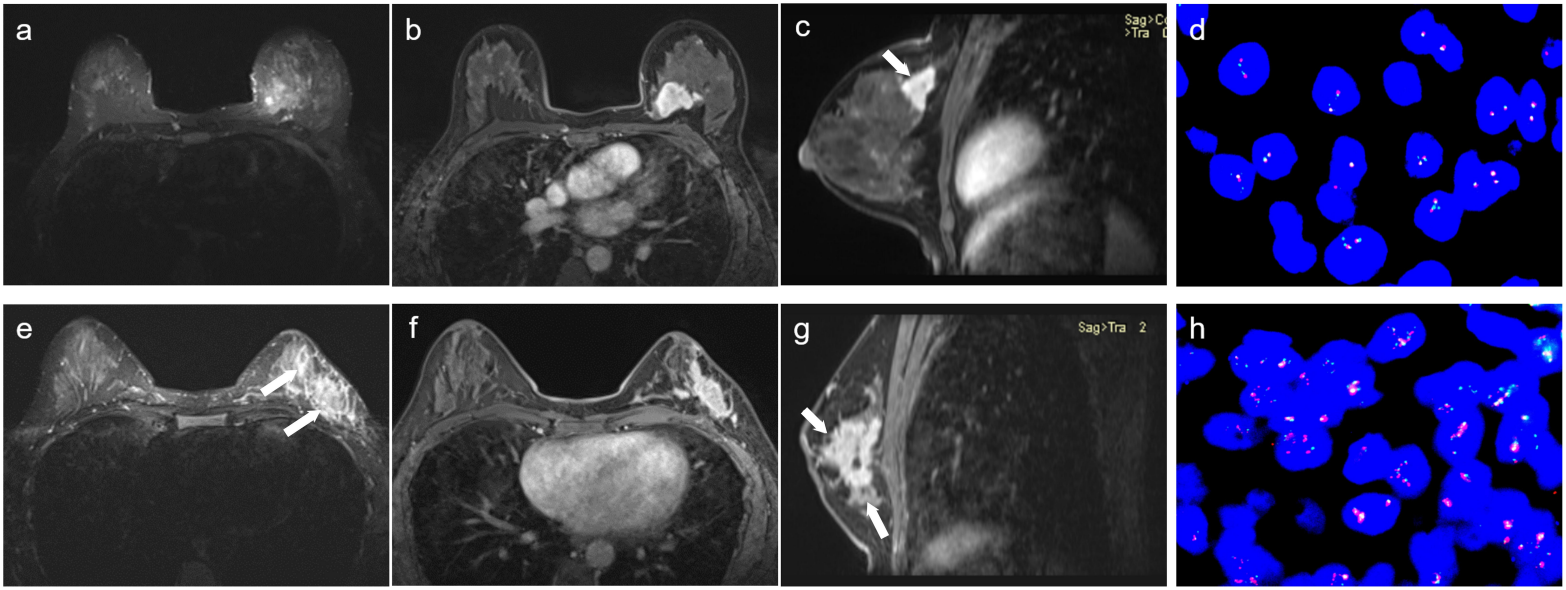
Representative multiparametric MRI images from the FISH-negative **(a–d)** and FISH-positive **(e–h)** groups. In the FISH-negative case **(d)**, T2WI **(a)** showed a hyperintense mass without peripheral edema (arrows). Axial **(b)** and sagittal **(c)** DCE_phase7_ images exhibited homogeneous enhancement with circumscribed margin (arrow). In contrast, the FISH-positive case **(h)** revealed a mass of mixed-intensity mass on T2WI **(e)**, accompanied by peripheral edema (arrows). Axial **(f)** and sagittal **(g)** DCE_phase7_ images showed heterogeneous enhancement and not circumscribed margin (arrows).

65 (60.75%) patients were FISH-negative (HER2-low) and 42 (39.25%) patients were FISH-positive (HER2-positive). In both the entire and training sets, Ki-67 level, tumor margin, and peripheral edema differed significantly between FISH-negative and FISH-positive patients. The other characteristics were not statistically significant across the three sets (**Tables 1**, **2**; **Supplementary Tables 1**, **2**). **Figure 3** showed the MRI characteristics of two IHC 2+ BC patients with different HER2 status.

**Table 1:** Clinical characteristics of IHC 2+ breast cancer patients in the training and testing sets.

Clinical characteristics	Training set (n=74)			Testing set (n=33)		
	FISH-negative (n=45)	FISH-positive (n=29)	p-value	FISH-negative (n=20)	FISH-positive (n=13)	p-value
Age (year), mean ± SD	53.27 ± 9.51	50.17 ± 9.53	0.176	52.35 ± 10.38	54.08 ± 9.69	0.635
ER, n (%)			0.462			0.659
Negative	11 (24.44%)	5 (17.24%)		3 (15.00%)	3 (23.08%)	
Positive	34 (75.56%)	24 (82.76%)		17 (85.00%)	10 (76.92%)	
PR n (%)			0.112			1.000
Negative	19 (42.22%)	7 (24.14%)		7 (35.00%)	4 (30.77%)	
Positive	26 (57.78%)	22 (75.86%)		13 (65.00%)	9 (69.23%)	
Ki-67 n (%)			0.025*			0.072
Negative	19 (42.22%)	5 (17.24%)		12 (60.00%)	3 (23.08%)	
Positive	26 (57.78%)	24 (82.76%)		8 (40.00%)	10 (76.92%)	
Histological grade n (%)			0.314			0.261
I	3 (6.67%)	1 (3.45%)		0 (0.00%)	0 (0.00%)	
II	34 (75.55%)	26 (89.66%)		17 (85.00%)	13 (100.00%)	
III	8 (17.78%)	2 (6.90%)		3 (15.00%)	0 (0.00%)	
Histological type n (%)			1.000			1.000
Non-special	44 (97.78%)	29 (100.00%)		18 (90.00%)	12 (92.31%)	
Special	1 (2.22%)	0 (0.00%)		2 (10.00%)	1 (7.69%)	

IHC, immunohistochemistry; FISH, fluorescence *in situ* hybridization; ER, estrogen receptor; PR, progesterone receptor.

**Table 2:** MRI characteristics of IHC 2+ breast cancer patients in the training and testing sets.

MRI characteristics	Training set (n=74)			Testing set (n=33)		
	FISH-negative (n=45)	FISH-positive (n=29)	p-value	FISH-negative (n=20)	FISH-positive (n=13)	p-value
Maximum diameter (cm), mean ± SD	2.48 ± 0.74	2.67 ± 1.18	0.392	2.61 ± 0.89	2.97 ± 1.24	0.342
Shape n (%)			0.107			0.472
Oval/round	33 (73.33%)	16 (55.17%)		13 (65.00%)	6 (46.15%)	
Irregular	12 (26.67%)	13 (44.83%)		7 (35.00%)	7 (53.85%)	
Margin n (%)			0.033*			0.296
Circumscribed	22 (48.89%)	7 (24.14%)		12 (60.00%)	5 (38.46%)	
Not circumscribed	23 (51.11%)	22 (75.86%)		8 (40.00%)	8 (61.54%)	
T2WI signal n (%)			0.539			0.517
Iso/hypointense	9 (20.00%)	8 (27.59%)		3 (15.00%)	3 (23.08%)	
Hyperintense	20 (44.44%)	14 (48.28%)		8 (40.00%)	7 (53.85%)	
Mixed-intensity	16 (35.56%)	7 (24.14%)		9 (45.00%)	3 (23.08%)	
Cystic necrosis n (%)			0.545			1.000
Yes	31 (68.89%)	18 (62.07%)		11 (55.00%)	7 (53.85%)	
No	14 (31.11%)	11 (37.93%)		9 (45.00%)	6 (46.15%)	
Peripheral edema n (%)			0.013*			0.08
Yes	24 (53.33%)	7 (24.14%)		13 (65.00%)	4 (30.77%)	
No	21 (46.67%)	22 (75.86%)		7 (35.00%)	9 (69.23%)	
DCEphase3 enhancement n (%)			0.181			0.071
Rim	10 (22.22%)	12 (41.38%)		2 (10.00%)	6 (46.15%)	
Heterogeneous	24 (53.33%)	13 (44.83%)		14 (70.00%)	6 (46.15%)	
Homogeneous	11 (24.44%)	4 (13.79%)		4 (20.00%)	1 (7.69%)	
DCEphase7 enhancement n (%)			0.102			0.326
Rim	22 (48.89%)	9 (31.03%)		6 (30.00%)	7 (53.85%)	
Heterogeneous	11 (24.44%)	14 (48.28%)		7 (35.00%)	4 (30.77%)	
Homogeneous	12 (26.67%)	6 (20.69%)		7 (35.00%)	2 (15.38%)	
TIC type n (%)			0.332			0.509
I (persistent)	2 (4.44%)	1 (3.45%)		1 (5.00%)	1 (7.69%)	
II (plateau)	25 (55.56%)	21 (72.41%)		12 (60.00%)	10 (76.92%)	
III (washout)	18 (40.00%)	7 (24.14%)		7 (35.00%)	2 (15.38%)	

IHC, immunohistochemistry; FISH, fluorescence *in situ* hybridization; T2WI, T2-weighted imaging; DCE_phase3_, the third postcontrast phase on dynamic contrast-enhanced T1-weighted imaging; DCE_phase7_, the seventh postcontrast phase on dynamic contrast-enhanced T1-weighted imaging; TIC, time-intensity curve.

### Feature selection and radiomics model

The radiomics features derived from T2WI, DWI, DCE_phase3_, and DCE_phase7_ demonstrated ICCs ranging from 0.19–0.99, 0.10–0.99, 0.08–0.99, and 0.13–0.99, respectively. Features with high stability and reproducibility (ICC > 0.75) were retained, yielding 1076, 1012, 1094, and 1132 features from each sequence. Following a rigorous feature selection process, 3 (T2WI), 5 (DWI), 4 (DCE_phase3_), and 5 (DCE_phase7_) features were selected to construct single-sequence radiomics models. Additionally, mpMRI radiomics models were built: model A (T2WI+DWI) with 8 features, model B (T2WI+DWI+DCE_phase3_) with 8 features, and model C (T2WI+DWI+DCE_phase7_) with 10 features. The features ultimately incorporated into each model were detailed in **Supplementary Table 3**.

### Model performance

**Figure 4:**
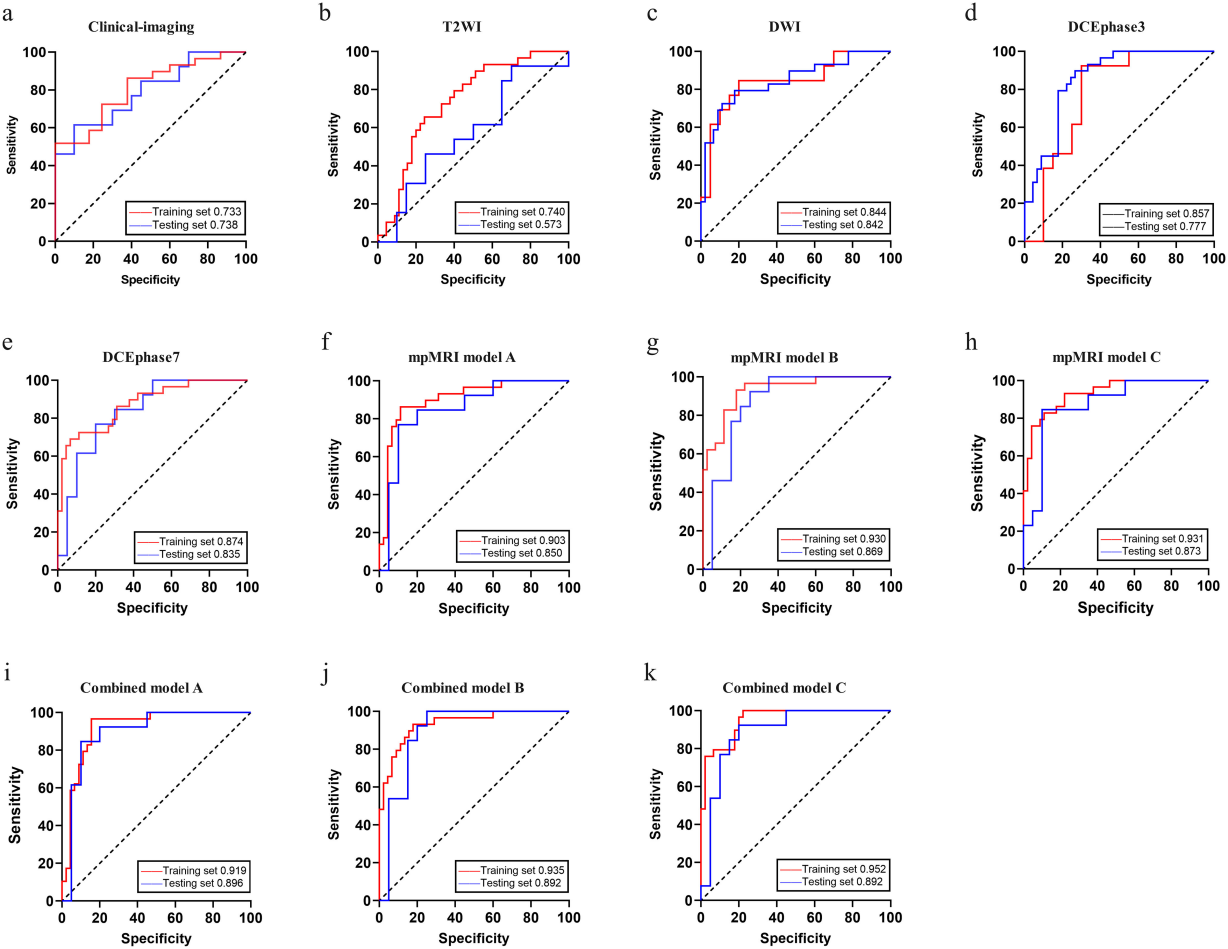
Receiver operating characteristic curves of different models **(a-k)** in the training and testing sets.

The predictive performance of different models was summarized in **Table 3** and **Figure 4**. The clinical-imaging model achieved AUCs of 0.733 [95% confidence interval (CI): 0.637-0.825] and 0.738 (95%CI: 0.577-0.879) in the training and testing sets, respectively. Among single-sequence-based radiomics models, the DCE_phase7_-based radiomics model performed best (training AUC: 0.874, 95%CI: 0.799-0.937; testing AUC: 0.835, 95%CI: 0.704-0.943), whereas the T2WI-based radiomics model performed worst (training AUC: 0.740, 95%CI: 0.639-0.835; testing AUC: 0.573, 95%CI: 0.394-0.736). In the training set, Delong test indicated no significant differences between other single-sequence models except between the DCE_phase7_ and T2WI models (p = 0.02). DCE_phase7_-based radiomics model had the highest specificity (93.3%) and accuracy (82.4%), and the DCE_phase3_-based radiomics model had the highest sensitivity (86.2%). In the testing set, Delong test indicated no significant differences between other single-sequence models except between the DWI and T2WI models (p = 0.02). DWI-based model had the highest sensitivity (84.6%), while the DCEphase7-based model had the highest specificity (80.0%); both shared the highest accuracy (78.8%).

**Table 3:** Predictive performance of different models based on radiomics features, clinical-imaging features, and their combinations.

Models	AUC (95%CI)	Sensitivity (%)	Specificity (%)	Accuracy (%)
Training set				
Clinical-imaging	0.733 (0.637, 0.825)	72.4%	62.2%	66.2%
T2WI	0.740 (0.639, 0.835)	62.1%	75.6%	70.3%
DWI	0.844 (0.760, 0.919)	75.9%	82.2%	79.7%
DCEphase3	0.857 (0.779, 0.922)	86.2%	73.3%	78.4%
DCEphase7	0.874 (0.799, 0.937)	65.5%	93.3%	82.4%
mpMRI model A	0.903 (0.839, 0.959)	82.8%	88.9%	86.5%
mpMRI model B	0.930 (0.875, 0.973)	89.7%	82.2%	85.1%
mpMRI model C	0.931 (0.878, 0.970)	79.3%	88.9%	85.1%
Combined model A	0.919 (0.861, 0.968)	93.1%	84.4%	87.8%
Combined model B	0.935 (0.882, 0.976)	89.7%	82.2%	85.1%
Combined model C	0.952 (0.911, 0.982)	96.6%	77.8%	85.1%
Testing set				
Clinical-imaging	0.738 (0.577, 0.879)	61.5%	70.0%	66.7%
T2WI	0.573 (0.394, 0.736)	46.2%	65.0%	57.6%
DWI	0.842 (0.703, 0.960)	84.6%	75.0%	78.8%
DCEphase3	0.777 (0.642, 0.904)	61.5%	75.0%	69.7%
DCEphase7	0.835 (0.704, 0.943)	76.9%	80.0%	78.8%
mpMRI model A	0.850 (0.722, 0.962)	76.9%	85.0%	81.8%
mpMRI model B	0.869 (0.758, 0.966)	92.3%	70.0%	78.8%
mpMRI model C	0.873 (0.752, 0.970)	76.9%	90.0%	84.8%
Combined model A	0.896 (0.791, 0.987)	84.6%	85.0%	84.8%
Combined model B	0.892 (0.790, 0.981)	84.6%	80.0%	81.8%
Combined model C	0.892 (0.780, 0.981)	92.3%	80.0%	84.8%

AUC, area under the curve; CI, confidence interval; T2WI, T2-weighted imaging; DWI, Diffusion-weighted imaging; DCE_phase3_, the third postcontrast phase on dynamic contrast-enhanced T1-weighted imaging; DCE_phase7_, the seventh postcontrast phase on dynamic contrast-enhanced T1-weighted imaging; mpMRI, multiparametric MRI.

**Figure 5:**
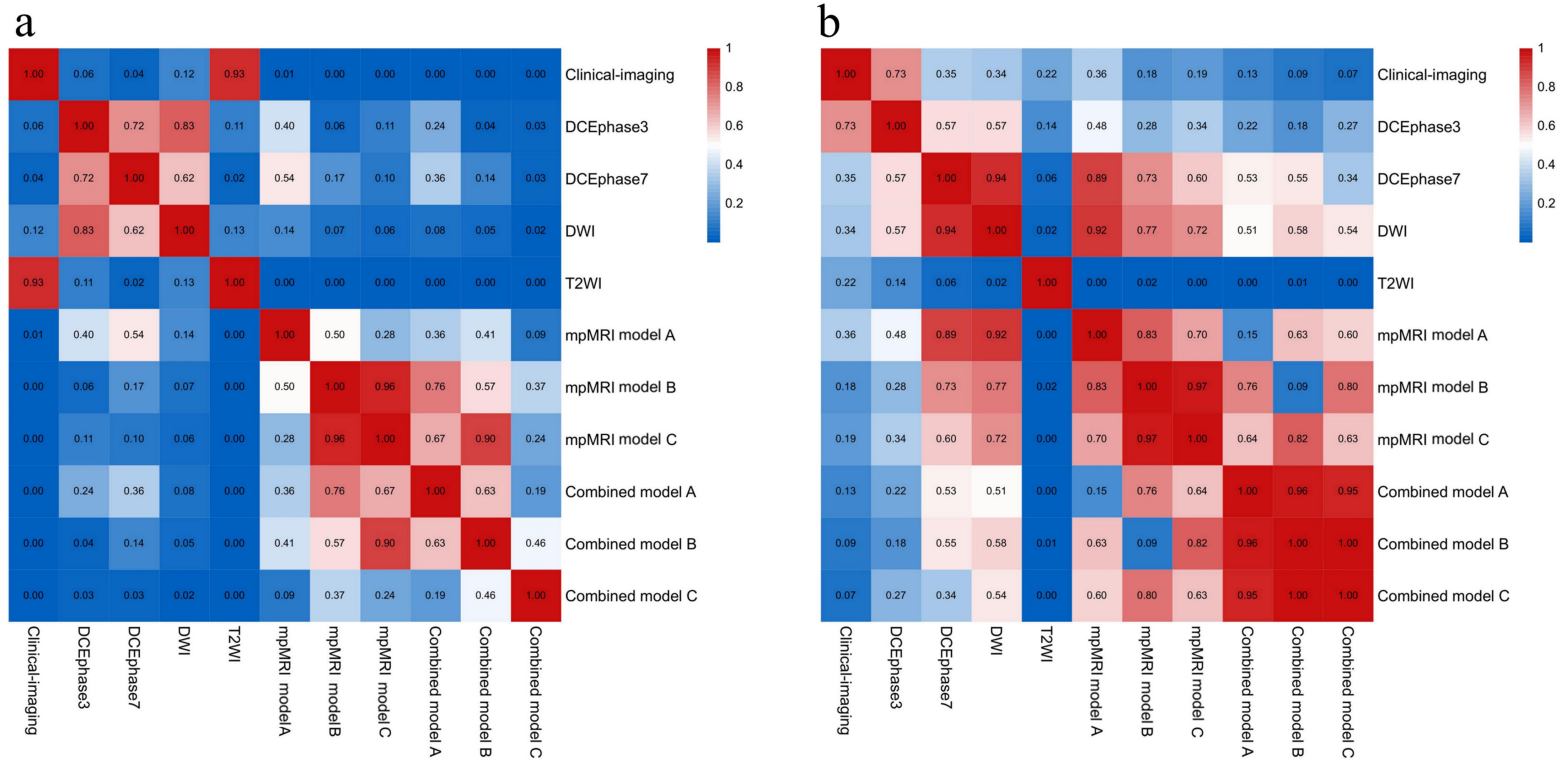
Statistical comparison of model performance using the DeLong test in the training **(a)** and testing **(b)** sets.

mpMRI-based radiomics models outperformed all single-sequence models, with AUCs further improving when fused with the clinical-imaging model. Combined model C achieved the highest training AUC (0.952, 95% CI: 0.911-0.982), with testing AUCs for combined models A-C being 0.896 (95%CI: 0.791-0.987), 0.892 (95%CI: 0.790-0.981), and 0.892 (95%CI: 0.780-0.981), respectively. In the training set, the DeLong test indicated significant differences in AUCs between the clinical-imaging model and each mpMRI model and combined model (p < 0.05). In the testing set, however, the AUC of each mpMRI model and combined model were higher than clinical-imaging model, but the differences were not statistically significant (p > 0.05). All inter-model AUC comparison p-values were shown in **Figure 5**.

## Discussion

This study demonstrates the feasibility and additive value of mpMRI-based radiomics in predicting HER2 status for breast cancer patients with equivocal (IHC 2+) results. By integrating radiomics features from both contrast-enhanced and non-contrast sequences, our combined models achieved superior diagnostic performance compared to conventional clinical-imaging assessment. The fusion of radiomics signatures with clinical-imaging features further enhanced predictive accuracy, highlighting the potential of this noninvasive approach as a complementary tool for refining HER2 status and guiding therapy.

Accurate determination of HER2 status is critical for treatment planning, as HER2 gene amplification or protein overexpression drives tumorigenesis and influences the use of targeted therapies such as trastuzumab (16, 17). In line with prior studies linking imaging features to HER2 status (18, 19), our analysis revealed that HER2-positive (FISH-positive) tumors were significantly associated with a higher Ki-67 index, not circumscribed margins, and peritumoral edema, and these features indicative of greater proliferative activity and biological aggressiveness (20). While a clinical-imaging model based on these characteristics showed moderate diagnostic value (training AUC: 0.733; testing AUC: 0.738), its limited accuracy underscored the need for more robust, noinvasive predictive tools.

Radiomics, which quantifies tumor heterogeneity through high-throughput feature extraction, offers a promising noninvasive and low-cost approach (12, 13). Prior studies have explored associations between radiomics features and HER2 status. For instance, Zhou et al. (21) reported that combining radiomics features from cranial-caudal and mediolateral oblique mammography views could effectively diagnose HER2 status, achieving an AUC of 0.846. Similarly, Li et al. (22) demonstrated that radiomics signature based on DCE-MRI predicted molecular subtypes of invasive BC, with an AUC of 0.65 for differentiating HER2-positive from HER2-negative. Our study focused on the feasibility of mpMRI-derived radiomics features for predicting HER2 status in IHC 2+ BC patients. Our results demonstrated that radiomics features extracted from DCE-MRI, DWI, and T2WI could differentiate FISH-negative from FISH-positive cases, further supporting the potential of mpMRI-based radiomics in BC.

DCE-MRI is widely used in radiomics due to its high spatial resolution and good signal-to-noise ratio. While DCE-MRI captures multiple postcontrast phases, analyses typically utilize a single phase (23, 24). In this study, radiomics features were extracted from two specific phases: DCE_phase3_, representing the initial peak enhancement with optimal tumor-to-tissue contrast (25); and DCE_phase7_, representing the last clearance phase where minimal enhancement. The DCE_phase3_-based radiomics model achieved AUCs of 0.857 in the training set and 0.777 in the testing set, consistent with prior work by Jiang et al. (26). To our knowledge, the delayed phase (DCE_phase7_) has not been previously explored for predicting HER2 status in IHC 2+ BC patients. Based on evidence that HER2-positive tumors exhibit faster contrast washout due to distinct vascular physiology (27), we hypothesized that DCE_phase7_ features would be informative. Our DCE_phase7_-based radiomics model demonstrated strong performance (training AUC: 0.874; testing AUC: 0.835), with numerically higher AUC, specificity, and accuracy than the DCE_phase3_ model, though not statistically significant.

DWI provides information on tissue microstructure and serves as a valuable complement to DCE-MRI for tumor characterization (28). Although DWI-based radiomics has shown potential in assessing receptor status in breast cancer (29), its application for predicting HER2 status, particularly in the IHC 2+ subgroup, has been less investigated. Given the reported association between HER2-positive and higher apparent diffusion coefficients (30), we hypothesized that radiomics could capture heterogeneity in tissue diffusivity between FISH-negative and FISH-positive groups. The resulting DWI-based model showed good discriminatory performance (training AUC: 0.844; testing AUC: 0.842), though slightly lower than the DCE-MRI models in the training set, possibly due to the lower spatial resolution of DWI.

T2WI depicts morphological features and the intrinsic tissue signals, which may correct with histological characteristics such as fibrosis, high proliferation cells, or necrosis. While these phenotypic differences suggest T2WI could hold discriminatory radiomics features, our T2WI-based radiomics model showed limited predictive performance (training AUC: 0.740; testing AUC: 0.573). The suboptimal result may be explained by the small number of robust features (n=3) ultimately selected from this sequence.

Both T2WI and DWI are routinely acquired non-contrast sequences. In our study, although their individual radiomics models underperformed relative to DCE-MRI models in the training set, their combination model (mpMRI model A) achieved superior predictive performance in both training and testing sets. This finding suggests that integrated radiomics features from non-contrast sequences offer a viable methodology for assessing HER2 status. Importantly, given DWI and T2WI sequences do not require contrast administration, this approach holds particular clinical relevance for patients with contraindications to gadolinium-based agents, such as contrast agent allergy or renal insufficiency.

mpMRI captures diverse tumor characteristics, including morphology, angiogenesis, cellularity, and microenvironment. In recent years, many researchers focused on mpMRI in BC, such as improving diagnostic accuracy (31) and predicting response to neoadjuvant therapy (32). In this study, integrating radiomics features from different sequences to construct mpMRI radiomics models (mpMRI model A: T2WI+DWI; B: T2WI+DWI+DCE_phase3_; C: T2WI+DWI+DCE_phase7_) consistently outperformed any single-sequence model. Furthermore, while the standalone clinical-imaging model was less predictive, its fusion with the mpMRI radiomics features, particularly with Model C, yielded the best overall performance (training AUC: 0.952; testing AUC: 0.892), demonstrating high sensitivity and accuracy.

This study has several limitations. First, its retrospective design and limited sample size may introduce selection bias. Second, as a single-center study utilizing only a 1.5T MRI scanner, the generalizability of our findings may be limited. External validation with larger, multi-center cohorts, particularly using 3.0T MRI systems, is therefore warranted. Third, our conventional MRI analysis focused on morphological characteristics, functional parameters, such as apparent diffusion coefficient and pharmacokinetic parameters, were not included.

## Conclusion

This study demonstrates that mpMRI-based radiomics models can effectively predict HER2 status in breast cancer patients with equivocal (IHC 2+) results. These models show superior performance compared to single-sequence radiomics or conventional clinical-imaging assessment, highlighting the value of integrating multiparametric imaging data as a noninvasive tool to distinguishing HER2 status.

## Data Availability

The raw data supporting the conclusions of this article will be made available by the authors, without undue reservation.
