# Supplementary material for: Multiparametric MRI radiomics for noninvasive prediction of HER2 status in immunohistochemical 2+ breast cancer
Source: Front Oncol. 2026 Jan 14;15:1765392. doi: 10.3389/fonc.2025.1765392 (PMC12846950; doi:10.3389/fonc.2025.1765392)
Supplement: Supplementary file 1 [file Table1.docx]

**Supplementary Table 1** Clinical characteristics of IHC 2+ breast cancer patients in the entire set

| Clinical characteristics | FISH | | p-value |
| --- | --- | --- | --- |
|  | Negative (n=65) | Positive (n=42) |  |
| Age (year), mean ± SD | 52.98±9.71 | 51.38±9.64 | 0.405 |
| ER, n(%) |  |  | 0.756 |
| Negative | 14(21.54%) | 8(19.05%) |  |
| Positive | 51(78.46%) | 34(80.95%) |  |
| PR n(%) |  |  | 0.142 |
| Negative | 26(40.00%) | 11(26.19%) |  |
| Positive | 39(60.00%) | 31(73.81%) |  |
| Ki-67 n(%) |  |  | 0.003* |
| Negative | 31(47.69%) | 8(19.05%) |  |
| Positive | 34(52.31%) | 34(80.95%) |  |
| Histological grade n(%) |  |  | 0.130 |
| I | 3(4.62%) | 1(2.38%) |  |
| II | 51(77.46%) | 39(92.86%) |  |
| III | 11(16.92%) | 2(4.76%) |  |
| Histological type n(%) |  |  | 0.942 |
| Non-special | 62(95.38%) | 41(97.62%) |  |
| Special | 3(4.62%) | 1(2.38%) |  |

Note: IHC, immunohistochemistry; FISH, fluorescence in situ hybridization; ER, estrogen receptor; PR, progesterone receptor.

**Supplementary Table 2** MRI characteristics of IHC 2+ breast cancer patients in the entire set

| MRI characteristics | FISH | | p-value |
| --- | --- | --- | --- |
|  | Negative (n=65) | Positive (n=42) |  |
| Maximum diameter (cm), mean ± SD | 2.52±0.79 | 2.76±1.19 | 0.205 |
| Shape n(%) |  |  | 0.054 |
| Oval/round | 46(70.77%) | 22(52.38%) |  |
| Irregular | 19(29.23%) | 20(47.62%) |  |
| Margin n(%) |  |  | 0.015* |
| Circumscribed | 34(52.31%) | 12(28.57%) |  |
| Not circumscribed | 31(47.69%) | 30(71.43%) |  |
| T2WI signal n(%) |  |  | 0.266 |
| Iso/hypointense | 12(18.46%) | 11(26.19%) |  |
| yperintense | 28(43.08%) | 21(50.00%) |  |
| Mixed-intensity | 25(38.46%) | 10(23.81%) |  |
| Cystic necrosis n(%) |  |  | 0.595 |
| Yes | 42(64.62%) | 25(59.52%) |  |
| No | 23(35.38%) | 17(40.48%) |  |
| Peripheral edema n(%) |  |  | 0.002* |
| Yes | 37(56.92%) | 11(26.19%) |  |
| No | 28(43.08%) | 31(73.81%) |  |
| DCE_phase3_ enhancement n(%) |  |  | 0.055 |
| Rim | 12(18.46%) | 16(38.10%) |  |
| Heterogeneous | 38(58.46%) | 21(50.00%) |  |
| Homogeneous | 15(23.08%) | 5(11.90%) |  |
| DCE_phase7_ enhancement n(%) |  |  | 0.229 |
| Rim | 28(43.08%) | 16(38.10%) |  |
| Heterogeneous | 18(27.69%) | 18(42.86%) |  |
| Homogeneous | 19(29.23%) | 8(19.05%) |  |
| TIC type n(%) |  |  | 0.177 |
| I (persistent) | 3(4.62%) | 2(4.76%) |  |
| II (plateau) | 37(56.92%) | 31(73.81%) |  |
| III (washout) | 25(38.46%) | 9(21.43%) |  |

Note: IHC, immunohistochemistry; FISH, fluorescence in situ hybridization; T2WI, T2-weighted imaging; DCE_phase3_, the third postcontrast phase on dynamic contrast-enhanced T1-weighted imaging; DCE_phase7_, the seventh postcontrast phase on dynamic contrast-enhanced T1-weighted imaging; TIC time-intensity curve.

**Supplementary Table 3** Radiomics features for different models

| Models | Features | Coefficients |
| --- | --- | --- |
| T2WI | Original_firstorder_Kurtosis | 0.6540 |
|  | Wavelet-HLL_firstorder_Median | 0.2094 |
|  | Wavelet-HHH_firstorder_Skewness | 0.6749 |
| DWI | Log-sigma-3-0-mm-3D_gldm_DependenceVariance | 0.5897 |
|  | Wavelet-LLH_glszm_HighGrayLevelZoneEmphasis | -0.6370 |
|  | Wavelet-LHH_firstorder_Kurtosis | 0.9263 |
|  | Wavelet-LHH_firstorder_Median | -1.1299 |
|  | Wavelet-LHH_firstorder_Skewness | 0.1207 |
| DCE_phase3_ | Original_firstorder_Minimum | -1.1130 |
|  | Wavelet-LLH_glcm_Imc1 | 1.3189 |
|  | Wavelet-HHH_glcm_Autocorrelation | -1.0180 |
|  | Wavelet-LLL_firstorder_Kurtosis | -0.9895 |
| DCE_phase7_ | Original_firstorder_Minimum | -1.4406 |
|  | Original_firstorder_Skewness | -0.2049 |
|  | Original_glcm_ClusterShade | -0.6186 |
|  | Wavelet-HLH_glcm_ClusterShade | -0.4653 |
|  | Wavelet-HLH_glszm_ZonePercentage | 0.7800 |
| mpMRI model A | DWI_log-sigma-2-0-mm-3D_glszm_SmallAreaHighGrayLevelEmphasis | -1.4062 |
|  | DWI_log-sigma-3-0-mm-3D_gldm_DependenceVariance | 0.4911 |
|  | DWI_wavelet-LLH_glszm_HighGrayLevelZoneEmphasis | -0.6037 |
|  | DWI_wavelet-LHH_firstorder_Kurtosis | 0.6414 |
|  | DWI_wavelet-LHH_firstorder_Median | -1.2165 |
|  | T2WI_wavelet-HLL_firstorder_Median | -0.4357 |
|  | T2WI_wavelet-HHH_firstorder_Skewness | 0.7730 |
|  | T2WI_wavelet-LLL_firstorder_Kurtosis | 0.6740 |
| mpMRI model B | DCE_phase3__wavelet-LLH_glcm_Imc1 | 1.3646 |
|  | DCE_phase3__wavelet-LLL_firstorder_Kurtosis | -1.7314 |
|  | DWI_original_glszm_SmallAreaHighGrayLevelEmphasis | -1.0021 |
|  | DWI_log-sigma-2-0-mm-3D_glszm_SmallAreaHighGrayLevelEmphasis | -1.5813 |
|  | DWI_wavelet-LLH_glrlm_HighGrayLevelRunEmphasis | 0.8528 |
|  | DWI_wavelet-LHL_glrlm_HighGrayLevelRunEmphasis | 0.7650 |
|  | T2WI_original_firstorder_Kurtosis | 1.4907 |
|  | T2WI_log-sigma-1-0-mm-3D_firstorder_Kurtosis | -0.2580 |
| mpMRI model C | DCE_phase7__original_firstorder_Minimum | -1.2154 |
|  | DCE_phase7__original_glcm_ClusterShade | -0.5031 |
|  | DCE_phase7__wavelet-LHH_glcm_ClusterShade | -1.0382 |
|  | DCE_phase7__wavelet-HLH_glszm_ZonePercentage | 0.5756 |
|  | DWI_log-sigma-2-0-mm-3D_glszm_SmallAreaHighGrayLevelEmphasis | -1.5144 |
|  | DWI_wavelet-LLH_glrlm_HighGrayLevelRunEmphasis | 0.6325 |
|  | DWI_wavelet-LHL_glrlm_HighGrayLevelRunEmphasis | -0.3159 |
|  | DWI_wavelet-LHH_firstorder_Kurtosis | 0.6410 |
|  | T2WI_log-sigma-1-0-mm-3D_firstorder_Kurtosis | 0.0980 |
|  | T2WI_wavelet-HHH_firstorder_Skewness | 0.6220 |

Note: T2WI, T2-weighted imaging; DWI, Diffusion-weighted imaging; DCE_phase3_, the third postcontrast phase on dynamic contrast-enhanced T1-weighted imaging; DCE_phase7_, the seventh post-contrast phase on dynamic contrast-enhanced T1-weighted imaging.
